# Predicted Structural Variability of *Mycobacterium tuberculosis* PPE18 Protein With Immunological Implications Among Clinical Strains

**DOI:** 10.3389/fmicb.2020.595312

**Published:** 2021-01-08

**Authors:** Jill M. C. Hakim, Zhenhua Yang

**Affiliations:** Department of Epidemiology, School of Public Health, University of Michigan, Ann Arbor, MI, United States

**Keywords:** immune evasion, *in silico* structural prediction, epitope prediction, PPE18, *Mycobacterium tuberculosis*

## Abstract

Recent advancements in vaccinology have led to the development of the M72/AS01E subunit vaccine, of which the major component is the *Mycobacterium tuberculosis* (MTB) PPE18 protein. Previous studies have demonstrated the genetic variability of the gene encoding PPE18 protein and the resulting peptide changes in diverse clinical strains of MTB; however, none have modeled the structural changes resulting from these peptide changes and their immunological implications. In this study, we investigated the structural predictions of 29 variant PPE18 proteins previously reported. We found evidence that PPE18 is at least a two-domain protein, with a highly conserved first domain and a largely variable second domain that has different coevolutionary clusters. Further, we investigated putative epitope sites in the clinical variants of PPE18 using prediction software. We found a negative relationship between T-cell epitope number and residue variability, while B-cell epitope likelihood was positively correlated with residue variability. Moreover, we found far more residues in the second domain predicted to be B-cell epitopes compared with the first domain. These results suggest an important functional role of the first domain and a role in immune evasion for the second, which extends our knowledge base of the basic biology of the PPE18 protein and indicates the need for further study into non-traditional immunological responses to TB.

## Introduction

*Mycobacterium tuberculosis* (MTB) is an ancient pathogen that is still responsible for a significant portion of global mortality and morbidity. Because of this, the World Health Organization and its global stop tuberculosis (TB) partners have set an ambitious goal to reduce the global incidence of TB to less than 100 per million population by 2035 (World Health Organization [WHO], 2015). To meet this goal, it is necessary to develop better tools for TB diagnosis, treatment, and vaccination (Raviglione and Sulis, 2016). The BCG vaccine, considered a vital tool in the arsenal against MTB, is approaching the 100th anniversary of its development, but despite its widespread use, BCG has major limitations in preventing adult MTB infection (Bisen et al., 2003). To improve and develop new tools to combat TB, there is a need for a better understanding of its causative pathogen.

Advancement in the fields of molecular biology and bioinformatics has enabled the use of *in silico* methods to answer questions that would have been difficult to address using traditional approaches. Recent applications of these methods have allowed investigations into predicted epitopes for antigenic MTB proteins, the evolution of antimicrobial genes, and rates of recombination in genes of interest (McNamara et al., 2010; Phelan et al., 2016). The PPE family of proteins, so named for the conserved PPE motif in the N terminal of their sequences, make up 7% of the MTB genome, and yet the function of this prevalent family is not well established (Brennan, 2017). Moreover, members of this family of proteins have been reported to have high levels of nucleotide diversity among clinical strains of MTB (Hebert et al., 2007; Vargas et al., 2019).

The PPE18 protein is of particular interest. The PPE18 protein has a high level of amino acid sequence variation among clinical isolates and is highly relevant both to vaccine development as well as to the basic pathogenesis of the disease (Homolka et al., 2016). *In vitro* studies have indicated the immune modulatory effect of the protein on the immune system, by interacting with TLR2 to illicit IL-10 production and dampening NFK-B response, overall dampening host inflammatory response (Udgata et al., 2016). In addition, the M72/AS01E vaccine, which completed phase 2b clinical trials and includes a recombinant PPE18, demonstrated 54% protection against active pulmonary TB (Van Der Meeren et al., 2018). Vaccinated individuals demonstrated immune responses 3 years after inoculation (Tait et al., 2019). Animal models have further demonstrated the efficacy of the M72/AS01 vaccine (Reed et al., 2003).

Despite PPE18’s potential as a new vaccine candidate, several studies have demonstrated a high level of sequence variability among clinical strains of global origins (Hebert et al., 2007; Homolka et al., 2016; Vargas et al., 2019). A PPE18 antigen encountered clinically that is different from that of the vaccine strain may not be recognized by the memory of immunity generated by PPE18 of the vaccine strain. This is important, not just for linear T-cell epitopes, but also for B-cell epitopes. Structural variability then becomes important when considering structural epitopes that the vaccine candidate might not illicit a response to. It is therefore important to investigate the structural impact and immunological implications of the reported non-synonymous sequence variations in PPE18 found among clinical isolates of MTB.

## Materials and Methods

### Study Dataset

This study used a total of 29 different protein sequences of PPE18 (Supplementary Material) that were identified among a total of 225 different clinical strains by Hebert et al. (2007) previously. These 225 strains, identified based on unique IS*6110* fingerprinting patterns (van Embden et al., 1993), represented a broad range of strains present in a population-based sample of 705 isolates collected in Arkansas between 1996 and 2000 and a hospital-based collection of 174 isolates from Turkey collected during 2000–2003. As described previously, the three principle genetic groups of MTB described by Sreevatsan et al. (1997) are represented in the 225 unique and clustered strains. In order to assess the representativeness of the PPE18 protein sequences analyzed in this study, we conducted a search of completed sequences of PPE18 in the NCBI protein database. We then compared the frequency of the most common residue between our modeled sequences and the sequences of 1,438 complete PPE18 sequences derived from the NCBI protein database.

### Protein Structure Prediction and Comparison

To expand upon previous research done on this protein, we investigated computationally predicted protein structures of the 29 PPE18 amino acid variants originally described by Hebert et al. Using the RaptorX web server, a template-based tertiary structure prediction software, and following the protocol previously described (Källberg et al., 2012), we predicted protein structures for each of the 29 PPE18 variants mentioned above as well as the MTB laboratory reference strain, H37RV.

To quantitatively compare the predicted PPE18 protein structures, we used TMalign to first find the best equivalent residues of each protein pair, and to then calculate the TM-score of each pair. TM-score measures the similarity of two protein structures and is more sensitive to global fold similarity than traditional root-mean-square deviation (Zhang and Skolnick, 2005). Pairwise analysis was visualized using the R corrplot package. We used several methods to compare amino acid variants and their predicted structures. Specifically, the alignment of amino acid variants was performed using the EMBL-EBI MUSCLE server, profile hidden Markov model analysis was done using HMMER, and sequence analysis was done using the BioPython package (Cock et al., 2009; Finn et al., 2011; Madeira et al., 2019).

### Evolution Analysis

To better define the variability profiles and understand the evolutionary background of the PPE18 protein, we investigated coevolutionary clusters among the study variants. Coevolution analysis was performed using BIS2, an openly accessible coevolution server that describes clusters of coevolving residues in peptide sequences (Oteri et al., 2017). Significantly predicted clusters with *P*-values <0.05 were used, and the number of maximal allowed exceptions admitted for capturing coevolutionary signals was between 0 and 1. Shannon’s entropy was used to quantify sequence variability. Shannon’s entropy measures the variability at a given position in the amino acid sequence and accounts for both the number of possible amino acids allowed at that position and their frequency.

### Epitope Prediction

To further understand the immunological impact of these structures in tandem with variability of the protein, we predicted epitopes using software available in the Immune Epitope Database (IEDB), MHCII binding prediction, and DiscoTope 2.0 (Kringelum et al., 2012). For T-cell epitope prediction, we used a set of MHCII DRB1 alleles that commonly occur in MTB endemic regions, as described previously (McNamara et al., 2010) and the recommended LC50 < 500 cutoff and the consensus algorithm (Kringelum et al., 2012). We used this specific set of alleles with the intention to generate data to pertinent populations carrying a high burden of TB that would most need a new TB vaccine. To define the T-cell epitope density metric, we first counted the number of times when each residue appeared in a unique epitope for each unique allele and then assigned to that residue a total epitope count. B-cell epitope prediction was done using DiscoTope 2.0, a software that predicts discontinuous structural B-cell epitopes using solvent accessibility and an epitope propensity amino acid score. We input each domain of the predicted H37RV structure to generate a list of propensity scores, indicating the likelihood for a given residue to be a part of a B-cell epitope. The propensity score cutoff chosen was the recommended -3.7 at which point the sensitivity and specificity of the prediction were 0.47 and 0.75, respectively (Kringelum et al., 2012).

## Results

### Predicted PPE18 Structures and Similarity Scores

**FIGURE 1:**
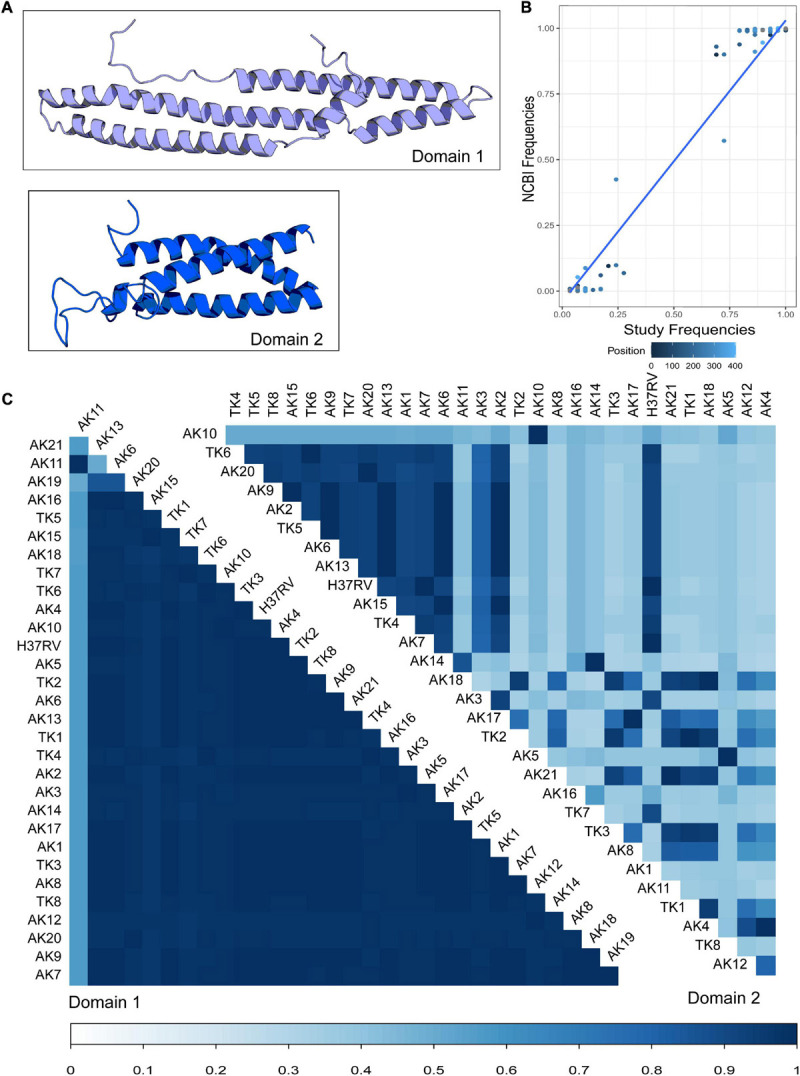
Modeling and comparison of *Mycobacterium tuberculosis* (MTB) protein PPE18 structures of clinical strains. **(A)** Predicted structures of two PPE18 protein domains for MTB laboratory reference strain H37Rv using RaptorX template-based tertiary structure prediction. **(B)** Comparison of the frequencies of the most common residue at each position in the PPE18 protein sequence between the 30 sequences modeled in this study and 1,438 complete PPE18 sequences obtained from the NCBI protein database. Each dot represents a given common residue between the two groups of sequences. Color indicates position in the sequence. Blue line shows a fitted GLM with *P-*value <2E-16. **(C)** Pairwise comparison of TM-align scores for variant strains investigated in this study. Variants AK1–AK21 are sequences found in Arkansas MTB strains. Variants TK1–TK8 represent sequences found in the Turkey MTB strains. A TM-align score of 0.3 and lower indicates random structural similarity. A TM-align score of 0.5 and higher indicates the same fold of similarity between the protein structures in comparison. Bottom bar indicates TM-align score.

Using the RaptorX modeling server, we generated predicted protein structures for the 29 study variants of PPE18 as well as a structure for the PPE18 in strain H37Rv, which was used as the reference for the analysis (Figure 1A). RaptorX predicted at least two separate protein domains, which were also predicted with Pfam and HMMER (Finn et al., 2011; Table 1). For all but five of the variant structures, the first domain ended at residue 179, the second at residue 298. Of exceptional note is variant AK19, which had a premature stop codon resulting in a truncated protein that contained only one predicted domain and had only a total peptide length of 154 residues.

**TABLE 1:** Frequency of *Mycobacterium tuberculosis* (MTB) PPE18 protein variants among 225 MTB clinical strains isolated in Turkey and Arkansas, United States, and the length of the first modeled domain for each variant.

**Variant**	**Variant frequency (%)**	**Domain 1 length**
H37RV	79.11	179
AK1	2.22	179
AK2	1.33	179
AK3	1.78	179
AK4	0.89	179
AK5	0.44	179
AK6	0.44	179
AK7	0.44	179
AK8	0.44	179
AK9	0.44	179
AK10	0.44	179
AK11	0.89	161
AK12	0.44	179
AK13	0.44	179
AK14	0.44	179
AK15	0.44	181
AK16	0.44	179
AK17	0.44	179
AK18	0.44	177
AK19	0.44	154
AK20	0.44	179
AK21	0.44	179
TK1	2.22	179
TK2	0.44	179
TK3	0.89	179
TK4	0.89	179
TK5	0.89	179
TK6	0.44	179
TK7	0.44	180
TK8	0.44	179

Using the predicted structure for each PPE18 variant, we attempted to quantify the structural variation between different variants. Using the Tm-Align server, we performed pairwise comparison of the structural similarity in each of the two predicted domains for all the study variants (Figure 1B; Zhang and Skolnick, 2005). In the first domain, most variants had largely similar structures, apart from variant AK11, and to a lesser extent, the truncated variant AK19. In the second domain, one group of variants clustered together in similarity, while variants outside of this group showed a high inter-variant variability.

To assess the representativeness of our study sample compared to a current population of MTB clinical strains, we compared the frequency of the most common residue at each locus between our modeled sequences and 1,438 complete PPE18 sequences derived from the NCBI protein database. This comparison demonstrated a strong linear relationship between the two groups of sequences, indicating that the majority residue is often represented in our dataset (Figure 1B).

### Level of Variability in Each Predicted Domain

**FIGURE 2:**
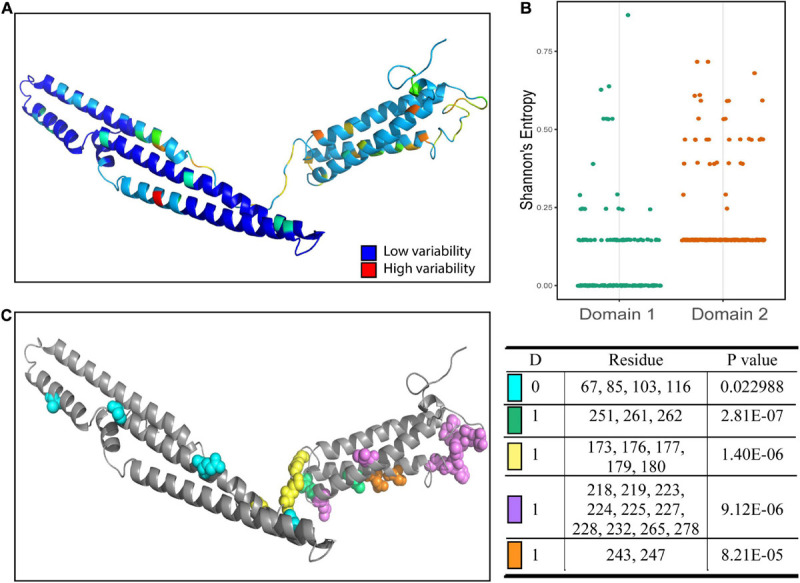
Amino acid sequence variability and coevolutionary clusters in each domain of PPE18. **(A)** Shannon’s entropy score mapped onto H37Rv reference strain. **(B)** Dot plot showing the Shannon’s entropy score of residues located in the first and second domains. A comparison between the mean entropy of the two groups using a two tailed unpaired Student’s t test resulted in a *P*-value <0.0001. **(C)** Coevolutionary clusters predicted by BIS2Analyzer. Clusters with *P*-value <0.05 as determined by Fisher’s exact test are shown. Each color represents residues that belong to the same coevolution cluster. The affiliated table indicates dimension **(D)**, which is the number of exceptions allowed for capturing coevolutionary signals, the residues included in each cluster, and the *P*-value for that cluster.

Different domains in proteins often perform distinct functions and different patterns of conservation of the proteins may arise. To better characterize the properties of the predicted domains, we used Shannon’s entropy to assess the variability at each residue. We found a significantly higher average level of variability for residues in the second domain compared to residues in the first domain (Figure 2A). The second domain had a higher overall level of variability, while much of the variability in the first domain was due to a few highly variable residues. To infer if these regions were functionally linked, we used BIS2Analyzer to determine coevolution between residues in the protein. We identified coevolved residues among the PPE18 variants and mapped these clusters onto the predicted H37Rv structure (Figure 2B). As expected, the variable regions within the first domain were coevolved, despite being distant from one another both in linear sequence and physical distance. Similarly, the coevolved clusters in the second domain were all confined to the second domain.

### Residue Variability and Epitope Prediction

**FIGURE 3:**
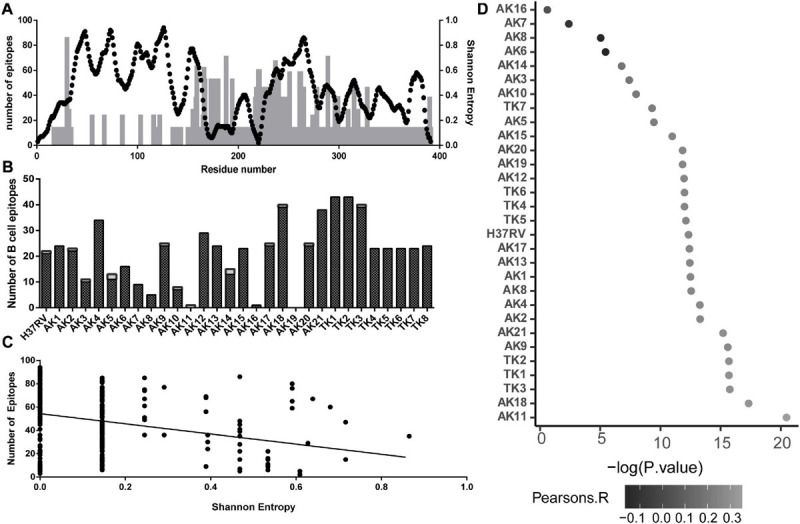
Association between sequence variability of PPE18 and the frequency of T- and B-cell epitopes for a select set of MHC II alleles that occur at high frequency in TB endemic countries, based on the analysis of all protein variants. **(A)** Illustration of T-cell epitope density and Shannon’s entropy score on a residue in the protein sequence. Black dots, number of epitopes; gray bars, Shannon’s entropy. **(B)** Number of B-cell epitopes per residue predicted by DiscoTope 2.0 on each variant for each domain. Black bars, domain one; white bar, domain 2. **(C)** Scatterplot demonstrating the relationship between number of T cell on a given residue and Shannon’s entropy of that residue. The line indicates the line of best fit, with a Pearson’s correlation -0.02294 and *P* < 0.0001. **(D)** Manhattan plot showing Pearson’s correlation between B-cell epitope score and Shannon’s entropy at each residue for all of the 30 predicted PPE18 structures. Pearson’s correlation is indicated by the grayscale value of the point, and X-axis represents the -log10 *P*-value.

Using the IEDB database of clinically validated T-cell epitopes and a consensus algorithm, we generated predicted T-cell epitope density on the multiple sequence alignment of all the variant sequences. This count was mapped onto the linear peptide sequence with the Shannon’s entropy at each residue (Figure 3A). We found a negative correlation between the number of T-cell epitopes containing each residue and Shannon’s entropy at that position (Figure 3B).

In addition to T-cell response, recent work has demonstrated the relevance of B-cell-mediated immune response in MTB infection (Counoupas and Triccas, 2019; Loxton, 2019). Using the predicted structures of the study variants of PPE18 protein, we found that the number of predicted B-cell epitopes for an individual variant structure ranged between 0 and 43 residues (Figure 3C). Consistent throughout all predicted structures was a higher proportion of possible B-cell epitopes in the second domain compared to that in the first domain. We then explored the possibility if evasion from B-cell-mediated immune recognition could be a potential source of sequence and structural variability. Using the H37Rv reference PPE18 protein structure, we found a highly significant relationship between Shannon’s entropy score and B-cell propensity score (Figure 3D). Because of the variation in the number of B-cell epitopes among different structural variants, despite the structural similarities in the first domain of the protein, we tested for this relationship in the B-cell epitope data calculated on each variant structure (Figure 3D). We found a statistically significant positive relationship between B-cell epitope propensity and variability at each residue for each variant structure excluding four variants. We also tested for an interaction between Shannon’s entropy and solvent accessibility for the H37RV structure to address the possibility that surface proteins were more likely to be mutated in a mechanism independent of B-cell evasion and found no significant relationship between entropy and solvent accessibility (*P* = 0.4945).

## Discussion

MTB PPE18 is highly relevant to the development of a new TB vaccine both due to its reported immunomodulatory role and being a major component in the candidate subunit vaccine M72/AS01E vaccine (Tait et al., 2019). As reported previously, the variability in the PPE18 gene could potentially impact the vaccine’s ability to illicit protection in a wide variety of people (Hebert et al., 2007; McNamara et al., 2010). Using template-based tertiary structure prediction software, we generated 30 models of PPE18 proteins, including that of the H37Rv reference strain and 29 protein amino sequence variants previously found in 225 clinical strains of MTB obtained from two geographic regions of the world.

The predicted structures revealed that PPE18 was at least a two-domain protein with a largely conserved first domain and a more variable second domain, both in structural and sequence similarity, across different PPE18 proteins found in diverse clinical strains. While previous studies have noted the similarity in the first 180 peptides among different PPE family proteins and subsequent variability in the following segments, this study demonstrates PPE18-specific inter-strain protein structural variability (Cole et al., 1998). Interestingly, the cluster of structural similarity revealed by the TM-align pairwise comparison in several variants’ second domain did not correspond to geographic source of the variant strain, indicating that these structural similarities may not be solely due to regional distribution. Due to the nature of this retrospective data, however, complete information on variant genetic lineage was missing, which limited our ability to explore the relationship between PPE18 structures and MTB genetic lineages. While a larger study with a more globally representative population, in terms of genetic lineages of the pathogen, would allow the identification of associations between clinical characteristics and protein variants, the present investigation has indicated a potentially interesting future direction.

The higher sequence and resultant structural conservation in the first domain of the PPE18 protein, compared with the second domain, recapitulates what is broadly known about PPE family proteins as a whole, but this study demonstrated the inter-strain variability of a specific member of the PPE family. These results could potentially indicate an important function that the first domain plays in the role of the protein as a whole; this possibility is further corroborated by the low structural variability in the first domain. This result aligns with previously published work by Nair et al. (2009) demonstrating that the interaction between PPE18 and TLR2 was dependent on the first 180 amino acids of the recombinant PPE18 protein. Moreover, the coevolution analysis demonstrated that some of the variable regions within the first domain were coevolved, indicating the importance of conservation within this domain. The non-contiguous nature of coevolved residues in this domain can also indicate a protein–protein interface (Oteri et al., 2017, p. 2; Rodriguez-Rivas et al., 2016). Although this study did not have host genetic information that would allow us to investigate this possibility further, this observation fits well with the hypothesis that this domain is involved with TLR2 interaction (Nair et al., 2009). An additional hypothetical protein–protein interface could be between PPE18 and proteins involved in the Type VII secretion system. These proteins involved in the Type VII secretion system have been reported to interact and be co-transcribed with other PPE family proteins in several previous studies (Gey van Pittius et al., 2006; Strong et al., 2006; Tundup et al., 2006). Several coevolved regions in the second domain also indicate some functional role in the second domain of this protein that is as of yet unknown.

Several studies have used computational approaches to investigate immunological epitope sites on antigenic sequences (Tomar and De, 2010; Potocnakova et al., 2016). Previous studies investigating HIV epitopes and Shannon’s entropy also found negative correlation between T-cell epitope number on each residue and the variability of that residue (Yusim et al., 2002; Allen et al., 2005). In addition, other studies have demonstrated high conservation in the CD4 and CD8 epitope regions of MTB (Comas et al., 2010; Coscolla et al., 2015). These findings suggest that MTB benefits from T-cell recognition and is under strong selection pressures to maintain conservation in epitope regions. In our study, we also observed a negative relationship between Shannon’s entropy and T-cell epitopes. However, because our work had several predicted structures of the PPE18 protein, we were able to take advantage of non-continuous B-cell epitope prediction software and investigated the relationship between the PPE18 variability and its possible B-cell epitopes. Leveraging this novel information, these results indicate a putative positive association between B-cell propensity score and variability, in contrast to the negative association between the possible T-cell epitopes and the variability of PPE18 protein. This could indicate that while PPE18 maintains conservation in T-cell epitope regions, it is under selection pressure that is moderately, although significantly, affected by humoral recognition. Using the modeled structures of each variant allowed the investigation of differential B-cell epitope predictions and demonstrated high variability in the number of predicted B-cell epitopes among PPE18 variants. This observation builds on work investigating heterogeneity of epitope recognition in MTB-infected individuals in different geographic regions (Valentini et al., 2017). These results are pertinent to novel vaccine development, as humoral immunity has gained increased interest in the battle against TB (Achkar et al., 2015; Niki et al., 2018). Especially relevant is a previous study demonstrating that a predicted open reading frame belonging to the PPE family could induce a strong B-cell response (Choudhary et al., 2003). This growing body of research, as well as our study, suggests further studies into humoral immunity triggered by PPE family proteins.

A major limitation of this work is the lack of empirical validation to further support inferences made from *in silico* modeling. While many of the software used in this study were accompanied with tests of significance that could give us some confidence in the validity of the models, predicted structures remain putative without *in vitro* assessment and validation of the predicted structures. Further clinical validation of these *in silico* results is important to further extend these inferences. In addition, while this study had study samples from geographically diverse populations, a larger population would have improved the generalizability of these results and allowed us to investigate clinical characteristics of MTB infection in tandem with structural variability profiles to help understand the biological consequences of the structural changes. Another limitation of the current study is that we have generated structures and completed the downstream analysis for a small number of variants; however, compared to a larger number of PPE18 amino acid variants found in 1,438 complete PPE18 sequences derived from the NCBI protein database, we demonstrate that the major residue was often shared between the two groups of proteins.

Despite these limitations, this study has numerous strengths. While structures resolved *in vitro* would have been preferred to modeled structures, the predicted structures agree with previously published literature (Nair et al., 2009). While the sample size was not large enough to make epidemiological inferences on the clinical characteristics of the various PPE18 structures present in different clinical strains, we were able to compare data from two geographically distinct cohorts.

## Conclusion

In conclusion, we have found that PPE18 is likely to be a protein with at least two domains, an inference further supported by distinct variability profiles for the two modeled domains, and the coevolution clusters found in each domain. While conservation of residues can indicate functional importance, residues that are commonly mutated together can also inform functional importance of the regions that those residues reside in Anishchenko et al. (2017). This could suggest functionally distinct domains whose functions must be conserved through non-random mutations. This study helps inform the basic biology of this important protein and potentially indicates directions of further study into non-traditional immunological responses to MTB.

## Data Availability Statement

The original contributions presented in the study are included in the article/Supplementary Material, further inquiries can be directed to the corresponding author/s.

## Author Contributions

JH contributed to the study design, conducted the data analysis, interpreted the study findings, and drafted and revised the manuscript. ZY conceived and designed the study, interpreted the study findings, and provided critical revision of the manuscript. Both authors reviewed and approved the final manuscript.

## Conflict of Interest

The authors declare that the research was conducted in the absence of any commercial or financial relationships that could be construed as a potential conflict of interest.
