# Supplementary material for: Predicted Structural Variability of Mycobacterium tuberculosis PPE18 Protein With Immunological Implications Among Clinical Strains
Source: Front Microbiol. 2021 Jan 8;11:595312. doi: 10.3389/fmicb.2020.595312 (PMC7819968; doi:10.3389/fmicb.2020.595312)
Supplement: Supplementary file 1 [file Table_1.DOCX]

>Reference

MVDFGALPPEINSARMYAGPGSASLVAAAQMWDSVASDLFSAASAFQSVVWGLTVGSWIGSSAGLMVAAASPYVAWMSVTAGQAELTAAQVRVAAAAYETAYGLTVPPPVIAENRAELMILIATNLLGQNTPAIAVNEAEYGEMWAQDAAAMFGYAAATATATATLLPFEEAPEMTSAGGLLEQAAAVEEASDTAAANQLMNNVPQALQQLAQPTQGTTPSSKLGGLWKTVSPHRSPISNMVSMANNHMSMTNSGVSMTNTLSSMLKGFAPAAAAQAVQTAAQNGVRAMSSLGSSLGSSGLGGGVAANLGRAASVGSLSVPQAWAAANQAVTPAARALPLTSLTSAAERGPGQMLGGLPVGQMGARAGGGLSGVLRVPPRPYVMPHSPAAG

>Variant5

MVDFGALPPEINSARMYAGPGSASLVAAAQMWDSVASDLFSAASAFQSVVWGLTVGSWIGSSAGLMVAAASPYVAWMSVTAGQAELTAAQVRVAAAAYETAYGLTVPPPVIAENRAELMILIATNLLGQNTPAIAVNEAEYGEMWAQDAAAMFGYAAATATATETLLPFEDAPLITNPGGLLEQAVAVEEAIDTAAANQLMNNVPQALQQLAQPTKSIWPFDQLSELWKAISPHLSPLSNIVSMLNNHVSMTNSGVSMASTLHSMLKGFAPAAAAEAVQTAAQNGVQAMSSLGSQGRSSLGSSGLGAGVAANLGRAASVGSLSVPQAWAAANQAVTPAARALPLTSLTSAAERGPGQMLGGLPVGQMGARAGGGLSGVLRVPPRPYVMPHSPAAG

>Variant16

MVDFGALPPEINSARMYAGPGSASLVAAAQMWDSVASDLFSAASAFQSVVWGLTVGSWIGSSAGLMVAAASPYVAWMSVTAGQAELTAAQVRVAAAAYETAYGLTVPPPVIAENRAELMILIATNLLGQNTPAIAVNEAEYGEMWAQDAAAMFGYAAATATATAALLPFEDAPLITNPGGLLEQAVAVEEAIDTAAANQLMNNVPQALQQLAQPTKSIWPFDQLSELWKAISPHLSPLSNIVSMLNNHVSMTNSGVSMASTLHSMLKGFAPAAAEAVQTAAQNGVQAMSSLGSSLGSSGLGGGVAANLGRAASVGSLSVPQAWAAANQAVTPAARALPLTSLTSAAERGPGQMLGGLPVGQMGARAGGGLSGVLRVPPRPYVIPHSPAAG

>Variant10

MVDFGALPPEINSARMYAGPGSASLVAAAQMWDSVASDLFSAASAFQSVVWGLTVGSWIGSSAGLMVAAASPYVAWMSVTAGQAELTAAQVRVAAAAYETAYGLTVPPPVIAENRAELMILIATNLLGQNTPAIAVNEAEYGEMWAQDAAAMFGYAATAATATEALLPFEDAPLITNPGGLLEQAVAVEEAIDTAAANQLMNNVPQALQQLAQPTKSIWPFDQLSELWKAISPHLSPLSNMVSMANNHMSMTNSGVSMTNTLHSMLKGFAPAAAAEAVQTAAQNGVRAMSSLGSQGRSSLGSSGLGGGVAANLGRAASVGSLSVPQAWAAANQAVTPAARALPLTSLTSAAERGPGQMLGGLPVGQMGARAGGGLSGVLRVPPRPYVMPHSPAAG

>Variant14

MVDFGALPPEINSARMYAGPGSASLVAAAQMWDSVASDLFSAASAFQSVVWGLTVGSWIGSSAGLMAAAASPYVAWMSVTAGQAQLTAAQVRVAAAAYETAYRLTVPPPVIAENRTELMTLTATNLLGQNTPAIEANQAAYSQMWAQDAEAMYYAAATAATATEALLPFEDAPLITNPGGLLEQAAAVEEASDTAAANQLMNNVPQALQQLAQPTQGTTPSSKLGGLWKTVSPHRSPISNMVSMANNHMSMTNSGVSMTNTLSSMLKGFAPAAAAQAVQTAAQNGVQAMSSLGSQGRSSLGSSGLGGGVAANLGRAASVGSLSVPQAWAAANQAVTPAARALPLTSLTSAAERGPGQMLGGLPVGQMGARAGGGLSGVLRVPPRPYVMPHSPAAG

>Variant17

MVDFGALPPEINSARMYAGPGSASLVAAAQMWDSVASDLFSAASAFQSVVWGLTVGSWIGSSAGLMVAAASPYVAWMSVTAGQAELTAAQVRVAAAAYETAYGLTVPPPVIAENRAELMILIATNLLGQNTPAIAVNEAEYGEMWAQDAAAMFGYAAAAATATATLLPFEEAPEMTSAGGLLEQAVAVEEASDTAAANQLMNNVPQALQQLAQPTQGTTPSSKLGGLWKTVSPHLSPLSNMVSMANNHVSMTNSGVSMASTLSSMLKGFAPAAAAQAVQTAAQNGVQAMSSLGSSLGSSGLGGGVAANSGRAASVGSLSVPQAWAAANQAVTPAARALPLTSLTSAAERGPGQMLGGLPVGQMGARAGGGLSGVLRVPPRPYVMPHSPAAG

>Variant 1

MVDFGALPPEINSARMYAGPGSASLVAAAQMWDSVASDLFSAASAFQSVVWGLTVGSWIGSSAGLMVAAASPYVAWMSVTAGQAELTAAQVRVAAAAYETAYGLTVPPPVIAENRAELMILIATNLLGQNTPAIAVNEAEYGEMWAQDAAAMFGYAAATATATATLLPFEEAPEMTSAGGLLEQAAAVEEASDTAAANQLMNNVPQALQQLAQPTQGTTPSSKLGGLWKTVSPHRSPISNMVSMANNHMSMTNSGVSMTNTLSSMLKGFAPAAAAQAVQTAAQNGVQAMSSLGSSLGSSGLGGGVAANLGRAASVGSLSVPQAWAAANQAVTPAARALPLTSLTSAAERGPGQMLGGLPVGQMGARAGGGLSGVLRVPPRPYVMPHSPAAG

>Variant3

MVDFGALPPEINSARMYAGPGSASLVAAAQMCDSVASDLFSAASAFQSVVWGLTVGSWIGSSAGLMVAAASPYVAWMSVTAGQAELTAAQVRVAAAAYETAYGLTVPPPVIAENRAELMILIATNLLGQNTPAIAVNEAEYGEMWAQDAAAMFGYAAATATATATLLPFEEAPEMTSAGGLLEQAAAVEEASDTAAANQLMNNVPQALQQLAQPTQGTTPSSKLGGLWKTVSPHLSPISNMVSMANNHMSMTNSGVSMTNTLSSMLKGFAPAAAAQAVQTAAQNGVQAMSSLGSSLGSSGLGGGVAANSGRAASVGSLSVPQAWAAANQAVTPAARALPLTSLTSAAERGPGQMLGGLPVGQMGARAGGGLSGVLRVPPRPYVMPHSPAAG

>Variant4

MVDFGALPPEINSARMYAGPGSASLVAAAQMWDSVASDLFSAASAFQSVVWGLTVGSWIGSSAGLMVAAASPYVAWMSVTAGQAELTAAQVRVAAAAYETAYGLTVPPPVIAENRAELMILIATNLLGQNTPAIAVNEAEYGEMWAQDAAAMFGYAAATATATATLLPFEEAPEMTSAGGLLEQAAAVEEASDTAAANQLMNNVPQALQQLAQPTQGTTPSSKLGGLWKTVSPHLSPISNMVSMANNHMSMTNSGVSMTNTLSSMLKGFAPAAAAQAVQTAAQNGVQAMSSLGSSLGSSGLGGGVAANSGRAASVGSLSVPQAWAAANQAVTPAARALPLTSLTSAAERGPGQMLGGLPVGQMGARAGGGLSGVLRVPPRPYVMPHSPAAG

>Variant8

MVDFGALPPEINSARMYAGPGSASLVAAAQMWDSVASDLFSAASAFQSVVWGLTVGSWIGSSAGLMVAAASPYVAWMSVTAGQAELTAAQVRVAAAAYETAYGLTVPPPVIAENRAELMILIATNLLGQNTPAIAVNEAEYGEMWAQDAAAMFGYAAATATATATLLPFEEAPEMTSAGGLLEQAAAVEEASDTAAANQLMNNVPQALQQLAQPTQGTTPSSKLGGLWKTVSPHRSPISNMVSMANNHMSMTNSGVSMTNTLSSMLKGFAPAAAAQAVQTAAQNGVQAMSSLGSQGRSSLGSSGLGGGVAANLGRAASVGSLSVPQAWAAANQAVTPAARALPLTSLTSAAERGPGQMLGGLPVGQMGARAGGGLSGVLRVPPRPYVMPHSPAAG

>Variant2

MVDFGALPPEINSARMYAGPGSASLVAAAKMWDSVASDLFSAASAFQSVVWGLTVGSWIGSSAGLMVAAASPYVAWMSVTAGQAELTAAQVRVAAAAYETAYGLTVPPPVIAENRAELMILIATNLLGQNTPAIAVNEAEYGEMWAQDAAAMFGYAAATATATATLLPFEEAPEMTSAGGLLEQAAAVEEASDTAAANQLMNNVPQALQQLAQPTQGTTPSSKLGGLWKTVSPHRSPISNMVSMANNHMSMTNSGVSMTNTLSSMLKGFAPAAAAQAVQTAAQNGVRAMSSLGSSLGSSGLGGGVAANLGRAASVGSLSVPQAWAAANQAVTPAARALPLTSLTSAAERGPGQMLGGLPVGQMGARAGGGLSGVLRVPPRPYVMPHSPAAG

>Variant9

MVDFGALPPEINSARMYAGPGSASLVAAAKMWDSVASDLFSAASAFQSVVWGLTVGSWIGSSAGLMVAAASPYVAWMSVTAGQAELTAAQVRVAAAAYETAYGLTVPPPVIAENRAELMILIATNLLGQNTPAIAVNEAEYGEMWAQDAAAMFGYAAATATATATLLPFEEAPEMTSAGGLLEQAAAVEEASDTAAANQLMNNVPQALQQLAQPTQGTTPSSKLGGLWKTVSPHRSPISNMVSMANNHMSMTNSGVSMTNTLSSMLKGFAPAAAAQAVQTAAQNGVRAMSSLGSSLGSSGLGGGVAANLGRADSVGSLSVPQAWAAANQAVTPAARALPLTSLTSAAERGPGQMLGGLPVGQMGARAGGGLSGVLRVPPRPYVMPHSPAAG

>Variant6

MVDFGALPPEINSARMYAGPGSASLVAAAQMWDSVASDLFSAASAFQSVVWGLTVGSWIGSSAGLMVAAASPYVAWMSVTAGQAELTAAQVRVAAAAYETAYGLTVPPPVIAENRAELMILIATNLLGQNTPAIAVNEAEYGEMWAQDAAAMFGYAAATATATATLLPFEEAPEMTSAGGLLEQAAAVEEASDTAAANQLMNNVPQALQQLAQPAQGVVPSSKLGGLWKTVSPHRSPISNMVSMANNHMSMTNSGVSMTNTLSSMLKGFAPAAAAQAVQTAAQNGVRAMSSLGSSLGSSGLGGGVAANLGRAASVGSLSVPQAWAAANQAVTPAARALPLTSLTSAAERGPGQMLGGLPVGQMGARAGGGLSGVLRVPPRPYVMPHSPAAG

>Variant12

MVDFGALPPEINSARMYAGPGSASLVAAAKMWDSVASDLFSAASAFQSVVWGLTVGSWIGSSAGLMAAAASPYVAWMSVTAGQAQLTAAQVRVAAAAYETAYRLTVPPPVIAENRTELMILIATNLLGQNTPAIAVNEAEYGEMWAQDAAAMFGYAAATATATATLLPFEEAPEMTSAGGLLEQAAAVEEAIDTAAANQLMNNVPQALQQLAQPAQGTTPSSKLGGLWKTVSPHLSPLSNMVSMANNHMSMTNSGVSMTNTLSSMLKGFAPAAAAQAVQTAAQNGVRAMSSLGSSLGSSGLGGGVAANLGRAASVGSLSVPQAWAAANQAVTPAARALPLTSLTSAAERGPGQMLGGLPVGQMGARAGGGLSGVLRVPPRPYVMPHSPAAG

>Variant20

MVDFGALPPEINSARMYAGPGSASLVAAAKMWDSVASDLFSAASAFQSVVWGLTVGSWIGSSAGLMVAAASPYVAWMSVTAGQAELTAAQVRVAAAAYETAYGLTVPPPVIAENRAELMILTATNLLGQNTPAIAVNEAEYGEMWAQDAAAMFGYAAATATATATLLPFEEAPEMTSAGGLLEQAVAVEEASDTAAANQLMNNVPQALQQLAQPTQGTTPSSKLGGLWKTVSPHRSPISNMVSMANNHMSMTNSGVSMTNTLSSMLKGFAPAAAAQAVQTAAQNGVRAMSSLGSSLGSSGLGGGVAANLGRAASVGSLSVPQAWAAANQAVTPAARALPLTSLTSAAERGPGQMLGGLPVGQMGARAGGGLSGVLRVPPRPYVMPHSPAAG

>Variant21

MVDFGALPPEINSARMYAGPGSASLVAAAQMWDSVASDLFSAASAFQSVVWGLTVGSWIGSSAGLMVAAASPYVAWMSVTAGQAELTAAQVRVAAAAYETAYGLTVPPPVIAENRAELMILIATNLLGQNTPAIAVNEAEYGEMWAQDAAAMFGYAAAAATATATLLPFEEAPEMTSAGGLLEQAVAVEEASDTAAANQLMNNVPQALQQLAQPTQGTTPSSKLGGLWKTVSPHRSPISNMVSMANNHMSMTNSGVSMTNTLSSMLKGFAPAAAAQAVQTAAQNGVRAMSSLGSSLGSSGLGGGVAANLGRAASVGSLSVPQAWAAANQAVTPAARALPLTSLTSAAERGPGQMLGGLPVGQMGARAGGGLSGVLRVPPRPYVIPHSPAAG

>Variant13

MVDFGALPPEINSARMYAGPGSASLVAAAKMWDSVASDLFSAASAFQSVVWGLTTGSWIGSSAGLMVAAASPYVAWMSVTAGQAELTAAQVRVAAAAYETAYGLTVPPPVIAENRAELMILIATNLLGQNTPAIAVNEAEYGEMWAQDAAAMFGYAAAAATATEALLPFEEAPEMTSAGGLLEQAAAVEEASDTAAANQLMNNVPQALQQLAQPTQGTTPSSKLGGLWKTVSPHRSPISNMVSMANNHMSMTNSGVSMTNTLSSMLKGFAPAAAAQAVQTAAQNGVRAMSSLGSSLGSSGLGGGVAANLGRAASVGSLSVPQAWAAANQAVTPAARALPLTSLTSAAERGPGQMLGGLPVGQMGARAGGGLSGVLRVPPRPYVMPHSPAAG

>Variant7

MVDFGALPPEINSARMYAGPGSASLVAAAKMWDSVASDLFSAASAFQSVVWGLTTGSWIGSSAGLMVAAASPYVAWMSVTAGQAELTAAQVRVAAAAYETAYGLTVPPPVIAENRAELMILIATNLLGQNTPAIAVNEAEYGEMWAQDAAAMFGYAAATATATATLLPFEEAPEMTSAGGLLEQAAAVEEASDTAAANQLMNNVPQALQQLAQPTQGTTPSSKLGGLWKTVSPHRSPISNMVSMANNHMSMTNSGVSMTNTLSSMLKGFAPAAAAQAVQTAAQNGVRAMSSLGSSLGSSGLGGGVAANLGRAASVGSLSVPQAWAAANQAVTPAARALPLTSLTSAAERGPGQMLGGLPVGQMGARAGGGLSGVLRVPPRPYVMPHSPAAG

>Variant15

MVDFGALPPEINSARMYAGPGSASLVAAAQMWDSVASDLFSAASAFQSVVWGLTVGSWIGSSAGLMVAAASPYVAWMSVTAGQAELTAAQVRVAAAAYETAYGLTVPPPVIAENRAELMILIATNLLGQNTPAIAVNEAEYGEMWAQDAAAMFGYAAATATAATTATLLPFEEAPEMTSAGGLLEQAAAVEEASDTAAANQLMNNVPQALQQLAQPTQGTTPSSKLGGLWKTVSPHRSPISNMVSMANNHMSMTNSGVSMTNTLSSMLKGFAPAAAAQAVQTAAQNGVRAMSSLGSSLGSSGLGGGVAANLGRAASVGSLSVPQAWAAANQAVTPAARALPLTSLTSAAERGPGQMLGGLPVGQMGARAGGGLSGVLRVPPRPYVMPHSPAAG

>Variant18

MVDFGALPPEINSARMYAGPGSASLVAAAQMWDSVASDLFSAASAFQSVVWGLTVGSWIGSSAGLMVAAASPYVAWMSVTAGQAELTAAQVRVAAAAYETAYGLTVPPPVIAENRAELMILIATNLLGQNTPAIAVNEAEYGEMWAQDAAAMFGYAAATATATLLPFEEAPEMTSAGGLLEQAAAVEEASDTAAANQLMNNVPQALQQLAQPTQGTTPSSKLGGLWKTVSPHRSPISNMVSMANNHMSMTNSGVSMTNTLSSMLKGFAPAAAAQAVQTAAQNGVRAMSSLGSSLGSSGLGGGVAANLGRAASVGSLSVPQAWAAANQAVTPAARALPLTSLTSAAERGPGQMLGGLPVGQMGARAGGGLSGVLRVPPRPYVMPHSPAAG

>Variant11

MVDFGALPPEINSARMVASDLFSAASAFQSVVWGLTVGSWIGSSAGLMVAAASPYVAWMSVTAGQAELTAAQVRVAAAAYETAYGLTVPPPVIAENRAELMILIATNLLGQNTPAIAVNEAEYGEMWAQDAAAMFGYAAATATATATLLPFEEAPEMTSAGGLLEQAAAVEEASDTAAANQLMNNVPQALQQLAQPTQGTTPSSKLGGLWKTVSPHRSPISNMVSMANNHMSMTNSGVSMTNTLSSMLKGFAPAAAAQAVQTAAQNGVRAMSSLGSSLGSSGLGGGVAANLGRAASVGSLSVPQAWAAANQAVTPAARALPLTSLTSAAERGPGQMLGGLPVGQMGARAGGGLSGVLRVPPRPYVMPHSPAAG

>Variant19

MVDFGALPPEINSARMYAGPGSASLVAAAQMWDSVASDLFSAASAFQSVVWGLTVGSWIGSSAGLMVAAASPYVAWMSVTAGQAELTAAQVRVAAAAYETAYGLTVPPPVIAENRAELMILIATNLLGQNTPAIAVNEAEYGEMWAQDAAAMFGX

>TK110

MVDFGALPPEINSARMYAGPGSASLVAAALMWDSVASDLFSAASAFQSVVWGLTVGSWIGSSAGLMVAAASPYVAWMSVTAGQAELTAAQVRVAAAAYETAYGLTVPPPVIAENRAELMILIATNLLGQNTPAIAVNEAEYGEMWAQDAAAMFGYAAATATATATLLPFEEAPEMTSAGGLLEQAAAVEEASDTAAANQLMNNVPQALQQLAQPTQGTTPSSKLGGLWKTVSPHRSPISNMVSMANNHMSMTNSGVSMTNTLSSMLKGFAPAAAAQAVQTAAQNGVRAMSSLGSSLGSSGLGGGVAANLGRAASVGSLSVPQAWAAANQAVTPAARALPLTSLTSAAERGPGQMLGGLPVGQMGARAGGGLSGVLRVPPRPYVMPHSPAAG

>TK112

MVDFGALPPEINSARMYAGPGSASLVAAALMWDSVASDLFSAASAFQSVVWGLTVGSWIGSSAGLMVAAASPYVAWMSVTAGQAELTAAQVRVAAAAYETAYGLTVPPPVIAENRAELMILIATNLLGQNTPAIAVNEAEYGEMWAQDAAAMFGYAAATATATATLLPFEEAPEMTSAGGLLEQAAAVEEASDTAAANQLMNNVPQALQQLAQPTQGTTPSSKLGGLWKTVSPHRSPISNMVSMANNHMSMTNSGVSMTNTLSSMLKGFAPAAAAQAVQTAAQNGVRAMSSLGSSLGSSGLGGGVAANLGRAASVGSLSVPQAWAAANQAVTPAARALPLTSLTSAAERGPGQMLGGLPVGQMGARAGGGLSGVLRVPPRPYVMPHSPAAG

>ZD073

MVDFGALPPEINSARMYAGPGSASLVAAAQMWDSVASDLFSAASAFQSVVWGLTVGSWIGSSAGLMVAAASPYVAWMSVTAGQAELTAAQVRVAAAAYETAYGLTVPPPVIAENRAELMILIATNLLGQNTPAIAVNEAEYGEMWAQDAAAMFGYAAAAATATATLLPFEEAPEMTSAGGLLEQAVAVEEASDTAAANQLMNNVPQALQQLAQPTQGTTPSSKLGGLWKTVSPHRSPISNMVSMANNHMSMTNSGVSMTNTLSSMLKGFAPAAAAQAVQTAAQNGVRAMSSLGSSLGSSGLGGGVAANLGRAASVGSLSVPQAWAAANQAVTPAARALPLTSLTSAAERGPGQMLGGLPVGQMGARAGGGLSGVLRVPPRPYVMPHSPAAG

>ZD053

MVDFGALPPEINSARMYAGPGSASLVAAAQMWDSVASDLFSAASAFQSVVWGLTVGSWIGSSAGLMVAAASPYVAWMSVTAGQAELTAAQVRVAAAAYETAYGLTVPPPVIAENRAELMILIATNLLGQNTPAIAVNEAEYGEMWAQDAAAMFGYAAATATATATLLPFEEAPEMTSAGGLLEQAVAVEEASDTAAANQLMNNVPQALQQLAQPTQGTTPSSKLGGLWKTVSPHRSPISNMVSMANNHVSMTNSGVSMASTLSSMLKGFAPAAAAQAVQTAAQNGVRAMSSLGSSLGSSGLGGGVAANLGRAASVGSLSVPQAWAAANQAVTPAARALPLTSLTSAAERGPGQMLGGLPVGQMGARAGGGLSGVLRVPPRPYVMPHSPAAG

>TK039

MVDFGALPPEINSARMYAGPGSASLVAAAQMWDSVASDLFSAASAFQSVVWGLTVGSWIGSSAGLMVAAASPYVAWMSVTAGQAELTAAQVRVAAAAYETAYGLTVPPPVIAENRAELMILIATNLLGQNTPAIAVNEAEYGEMWAQDAAAMFGYAAATATATATLLPFEEAPEMTSAGGLLEQAAAVEEASDTAAANQLMNNVPQALQQLAQPTQGTTPSSKLGGLWKTVSPHRSPISNMVSMANNHVSMTNSGVSMASTLSSMLKGFAPAAAAQAVQTAAQNGVRAMSSLGSSLGSSGLGGGVAANLGRAASVGSLSVPQAWAAANQAVTPAARALPLTSLTSAAERGPGQMLGGLPVGQMGARAGGGLSGVLRVPPRPYVMPHSPAAG

>TK013

MVDFGALPPEINSARMYAGPGSASLVAAAQMWDSVASDLFSAASAFQSVVWGLTVGSWIGSSAGLMVAAASPYVAWMSVTAGQAELTAAQVRVAAAAYETAYGLTVPPPVIAENRAELMILIATNLLGQNTPAIAVNEAEYGEMWAQDAAAMFGYAAATATATATLLPFEEAPEMTSAGGLLEQAAAVEEASDTAAANQLMNNVPQALQQLAQPTQGTTPSSKLGGLWKTVSPHRSPISNMVSMANNHMSMTNSGVSMTNTLSSMLKGFAPAAAAQAVQTAAQNGVRAMSSLGSSLGSSGLGGGVAANLGRAASVGSLSVPPAWAAANQAVTPAARALPLTSLTSAAERGPGQMLGGLPVGQMGARAGGGLSGVLRVPPRPYVMPHSPAAG

>TK016

MVDFGALPPEINSARMYAGPGSASLVAAAQMWDSVASDLFSAASAFQSVVWGLTVGSWIGSSAGLMVAAASPYVAWMSVTAGQAELTAAQVRVAAAAYETAYGLTVPPPVIAENRAELMILIATNLLGQNTPAIAVNEAEYGEMWAQDAAAMFGYAAATATATATLLPFEEAPEMTSAGGLLEQAAAVEEASDTAAANQLMNNVPQALQQLAQPTQGTTPSSKLGGLWKTVSPHRSPISNMVSMANNHMSMTNSGVSMTNTLSSMLKGFAPAAAAQAVQTAAQNGVRAMSSLGSSLGSSGLGGGVAANLGRAASVGSLSVPPAWAAANQAVTPAARALPLTSLTSAAERGPGQMLGGLPVGQMGARAGGGLSGVLRVPPRPYVMPHSPAAG

>TK065

MVDFGALPPEINSARMYAGPGSASLVAAAQMWDSVASDLFSAASAFQSVVWGLTVGSWIGSSAGLMVAAASPYVAWMSVTAGQAELTAAQVRVAAAAYETAYGLTVPPPVIAENRAELMILIATNLLGQNTPAIAVNEAEYGEMWAQDAAAMFGYAAATATATATLLPFEEAPEMTSAGGLLEQAAAVEEASDTAAANQLMNNVPQALQQLAQPTQGTTPSSKLGGLWKTVSPHRSPISNMVSMANNHMSMTNSGVSMTNTLSSMLKGFAPAAAAQAVQTAAQNGVRAMSSLGSSLGSSGLGGGVAANLGRAASVGSLSVPPAWAAANQAVTPAARALPLTSLTSAAERGPGQMLGGLPVGQMGARAGGGLSGVLRVPPRPYVMPHSPAAG
